# High‐intensity training as a novel treatment for impaired awareness of hypoglycaemia in type 1 diabetes [HIT4HYPOS]: Protocol for a randomized parallel‐group study

**DOI:** 10.1002/edm2.166

**Published:** 2020-07-21

**Authors:** Catriona M. Farrell, Alison D. McNeilly, Daniel West, Rory J. McCrimmon

**Affiliations:** ^1^ Division of Systems Medicine School of Medicine University of Dundee Dundee UK; ^2^ Institute of Cellular Medicine Faculty of Medical Science Newcastle University Newcastle UK

**Keywords:** adrenaline, behaviour, counterregulation, diabetes, exercise, habituation, hypoglycaemia, impaired awareness

## Abstract

**Aim:**

This pilot study aimed to investigate whether a 4‐week programme of intermittent high‐intensity training (HIT) will improve counterregulatory responses and improve hypoglycaemia awareness in adults with type 1 diabetes who have been exposed to recurrent hypoglycaemia.

**Methods:**

Adults with type 1 diabetes who have been exposed to recurrent hypoglycaemia will be recruited from NHS Tayside, Scotland. All participants have a 4‐week run‐in period to optimize glycaemic control and to receive instruction in hypoglycaemia avoidance using insulin dose adjustment and real‐time continuous glucose monitoring (CGM). Following this, they will undergo a baseline 90‐minute hyperinsulinaemic hypoglycaemic clamp to assess symptomatic, cognitive and hormonal counterregulatory responses. Subsequently, participants will be randomized in a parallel‐group design to either undergo a 4‐week intervention with HIT or to no exercise with both groups using CGM throughout and receiving additional advice on hypoglycaemia avoidance. Participants in the HIT arm of the trial will be instructed to exercise 3 times a week on a cycle ergometer and asked to achieve ≥ 90% max heart rate during each period of exercise. On completion of the intervention period, all subjects then undergo a second matched hyperinsulinaemic hypoglycaemic clamp study.

**Discussion:**

This pilot study will determine whether high‐intensity exercise may offer a novel approach to restore hypoglycaemic awareness in type 1 diabetes (International Standard Randomised Controlled Trials No: ISRCTN15373978).

## Graphical abstract

A single episode of high intensity training has been shown to at least temporarily improve hormonal, symptomatic and cognitive responses to hypoglycaemia in people with type 1 diabetes and impaired awareness of hypoglycaemia (IAH) through a process referred to as dishabituation. The current study has been designed to examine whether this effect is sustained after a 4‐week high intensity exercise programme when compared to use of continuous glucose monitoring, the current gold standard of treatment for IAH.

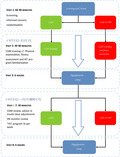

## 1 INTRODUCTION

Impaired awareness of hypoglycaemia (IAH) is defined as ‘a diminished ability to perceive the onset of acute hypoglycaemia’.^1^ IAH affects 20%‐25% of all people with type 1 diabetes, and of concern, the incidence of IAH has not changed in the last 2‐3 decades despite the introduction of insulin analogues and improved insulin delivery systems.^2^ In people with type 1 diabetes, IAH increases the risk of severe hypoglycaemia (defined as the need for external assistance to recover) by up to sixfold.^3^ IAH is an acquired abnormality that is essentially a complication of insulin therapy; it should be placed alongside retinopathy, neuropathy and nephropathy as its morbidity and mortality can be just as serious and disabling. The underlying mechanisms that lead to the development of IAH are unclear; however, it has been well established in multiple studies in people with and without type 1 diabetes that IAH can develop by inducing one or more episodes of experimentally induced hypoglycaemia.^4^ In addition, the greater the frequency of incidence exposure to hypoglycaemia, the greater the degree of suppression of symptomatic and hormonal counterregulatory responses (CRR), a process that can be reversed through strict hypoglycaemia avoidance.^5^, ^6^, ^7^

Habituation is a form of adaptive memory that develops in many organisms in response to a repeated, often stressful stimulus.^8^ Habituation is defined as a ‘reduction of the psychological, behavioural or physiological response to a stimulus as a result of repeated or prolonged exposure’.^8^, ^9^ This definition of a habituated response is consistent with the changes in CRR evoked by repeated hypoglycaemia in people with type 1 diabetes where there is also a progressive reduction or loss of psychological (anxiety), behavioural (food seeking) and physiological (symptom and hormonal) CRR.^8^, ^9^, ^10^ This suggests the hypothesis that IAH develops through habituation to repeated hypoglycaemia. To test this hypothesis, we recently conducted two proof‐of‐concept studies in rodents^11^ and humans.^12^ In these two studies, we sought to determine whether IAH could be at least temporarily improved by the introduction of a novel stress stimulus, a phenomenon referred to as dishabituation and a key feature of habituation. Consistent with our hypothesis, we reported that a single 20‐minute programme of high‐intensity exercise significantly augmented the CRR to subsequent hypoglycaemia in both the rodent model^11^ and humans with long‐duration type 1 diabetes and IAH.^12^ However, while we have demonstrated that a single episode of HIT can at least temporarily improve the counterregulatory defect induced by recurrent hypoglycaemia, it remains possible that the effect is (a) not sustained or (b) individuals may over time adapt to the novel dishabituating stimulus.

## 2 AIM

To investigate whether a 4‐week programme of intermittent high‐intensity exercise will improve counterregulatory responses and restore hypoglycaemia awareness in adults with T1D and IAH beyond the benefits that may be achieved through optimal treatment.^13^

## 3 PARTICIPANTS AND METHODS

### 3.1 Study design

This is a randomized, parallel‐group, single‐centre pilot study conducted in NHS Tayside, Scotland. Up to 32 participants will be randomized 1:1 to intervention; 4‐week programme of intermittent high‐intensity exercise and continuous glucose monitoring, or control; and 4 weeks of continuous glucose monitoring alone.

### 3.2 Study population

- Impaired awareness of hypoglycaemia (Gold score ≥4 or Modified Clark score ≥4 or DAFNE hypoglycaemia awareness rating 2 or 3) and/or evidence of recurrent hypoglycaemia (defined as ≥2 blood glucose or flash glucose readings of <4.0 mmol/L a week, based on long‐term history and up to 90 days flash glucose monitoring or CGM).

Inclusion criteria were as follows:

- Adults ≥18 and ≤55 years
- Type 1 diabetes
  - >5 years' disease duration
  - HbA1c < 75 mmol/L
- On intensive insulin therapy (CSII or MDI)

Exclusion criteria were as follows:

- Competitive sportsman/woman
- History of significant heart disease
- Ischaemic heart disease, congestive cardiac failure or cardiac surgery
- Treatment with beta‐blockers
- Treatment with oral steroids within the last 6 months
- Anaemia (Hb < 120 g/L for women, Hb < 130 g/L for men)
- Renal impairment (eGFR < 60)
- History of significant lung disease—that limits exercise
- History of significant neurological disease—those with a history of seizures second to hypoglycaemia must be seizure‐free for 12 months prior to consent.
- High‐risk foot disease
- Previous amputation of toes/foot/leg
- Pregnant women or breastfeeding mothers
- Participation in HIT or equivalent in past 6 months
- Physical ability that may limit exercise
- Inability to give consent

## 4 METHODS

### 4.1 Screening

Potential participants will be identified using the Scottish Diabetes Research Network (SDRN) and from the diabetes outpatient clinics in NHS Tayside, Scotland. They will be invited to attend a screening visit where informed written consent will be given and eligibility confirmed. This will involve a review of their medical history, recent blood results and flash glucose monitoring if applicable and completion of three questionnaires: Gold et al,^14^ Clarke et al^15^ and DAFNE,^16^ to determine degree of subjective hypoglycaemia awareness.

**FIGURE 1:**
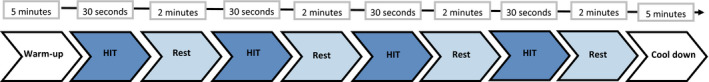
High‐intensity training programme

**FIGURE 2:**
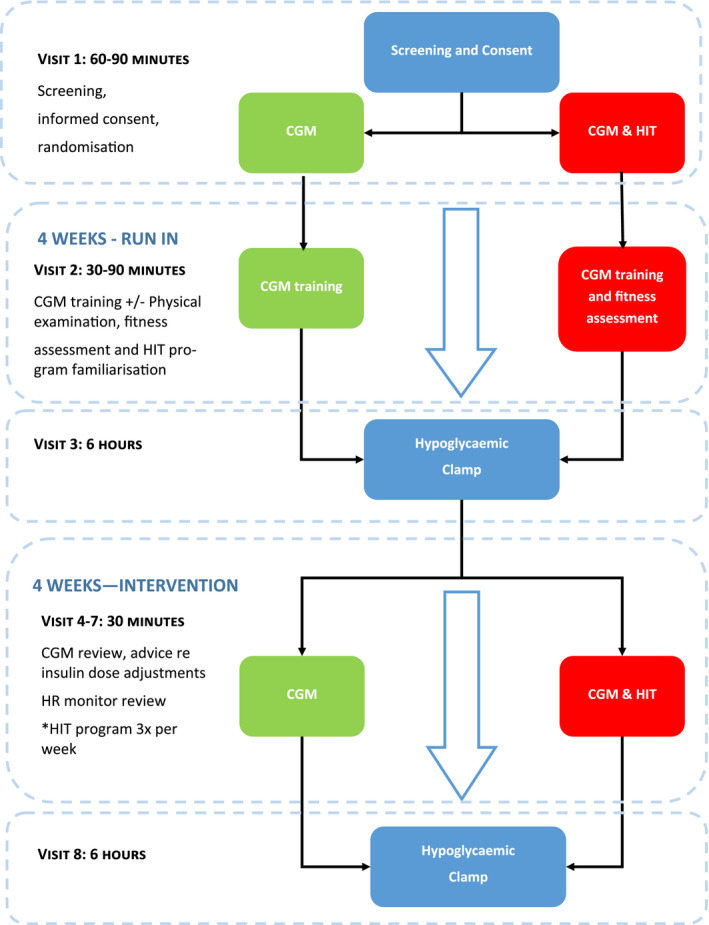
Study flow diagram

After entering the study, participants will be randomized to either the HIT programme (Figure 1) along with CGM (HIT&CGM) or CGM alone (control), in conjunction with advice on hypoglycaemia avoidance (Figure 2).

### 4.2 Run‐in period

All participants will then enter an initial 4‐week run‐in period. During this time, their glycaemic control will be optimized through insulin dose adjustment, review of carbohydrate counting/ratios and instructions on hypoglycaemia avoidance. This will be done via a phone call or email once a week by the Clinical Research Fellow (CRF).

During the 4‐week run‐in period, there will be one study visit (visit 2), which takes place in week 1 or 2. During this visit, all participants will be taught how to fit the real‐time continuous glucose monitor (CGM) (Dexcom G6; Dexcom) and shown how it works.

For those participants in the HIT&CGM group, during visit 2 medical history will be detailed and participants will undergo clinical examination. An electrocardiogram and vital signs including blood pressure and heart rate will be checked as a safety measure. Participants will undergo an assessment of their baseline fitness by carrying out a VO_2_ peak and heart rate peak assessment on a cycle ergometer (Corival, Lode; Metamax 3B, CORTEX Biophysik). The VO_2_ peak will be performed as an incremental maximum fitness test; 2 minutes at rest to ensure accurate readings followed by 5‐minute warm‐up at 25w, cycling at a cadence of 40‐60RPM. The incremental protocol will then begin at 35w, increasing by 15w per minute while maintaining a cadence of approximately 60RPM. Rating of perceived exertion (RPE) will be assessed every minute during the test using the Borg scale.^17^ The test will continue until VO_2_ peak is reached. Three of the following four criteria need to be satisfied:

- RPE ≥ 18
- Respiratory exchange ratio >1.1
- Volitional fatigue
- Maximum estimated heart rate (220‐age)

The peak heart rate reached will be used to design the HIT programme for each participant. Participants will be given standard advice regarding insulin dose adjustments prior to and following the VO_2_ peak assessment.^18^

This visit will take place during the first 2 weeks of the run‐in period to allow any effect from VO_2_ max and heart rate peak assessment to have worn off.

### 4.3 Experimental Hypoglycaemia: Hyperinsulinaemic hypoglycaemic clamp study

At the end of the 4‐week run‐in period, participants will return to the Clinical Research Centre for visit 3 where they will undergo a hyperinsulinaemic hypoglycaemic clamp.^19^ They will have been fasted from mid‐night (drinking clear fluids only). CGM data will first be analysed to ensure no significant hypoglycaemia (<3.0 mmol/L for ≥20 minutes)^20^, ^21^ in the preceding 24 hours. If there is evidence of hypoglycaemia, the study will be postponed and the visit repeated. If there is no evidence of hypoglycaemia, each participant will then undergo a controlled 90‐minute hyperinsulinaemic hypoglycaemic (2.5 mmol/L) clamp study.

In this procedure, insulin (0.3 U/mL) is infused at 50 mL/h for priming purposes until the blood glucose drops to below 7 mmol/L, after which a rate of 1.5 mU/kg/min will be maintained for the duration of the clamp. 20% dextrose is simultaneously infused at a variable rate (Infusomat Space; B. Braun Medical Ltd) based on frequent (every 5 minutes) bedside plasma glucose measures (Biosen C‐Line GP+; EKF diagnostics), to maintain blood glucose at predetermined levels. In this study, euglycaemia (glucose target 5.0 mmol/L) is initially achieved and maintained for the first 30 minutes of the glucose clamp study, and subsequently, blood glucose will be reduced over 30 minutes to a target plasma glucose level of 2.5 mmol/L, which is then maintained for a further 60 minutes before glucose levels are returned to the euglycaemic range.

Blood samples will be taken every 30 minutes during the hyperinsulinaemic euglycaemic and hypoglycaemic clamp (*t* = −30, 0, 30, 60, 90 minutes). Glucose, lactate, glucagon, adrenaline, noradrenaline, insulin and cortisol will be measured.

In addition, at each 30‐minute time point (*t* = −30, 0, 30, 60, 90 minutes) participants' cognitive function will be assessed using the Digit symbol substitution test (DSST^22^) and 4‐choice reaction time test (4CRT^23^). The Edinburgh Hypoglycaemia Symptom (EHS^24^) score will be used to assess symptom awareness at each of these time points. At the start of the hyperinsulinaemic hypoglycaemic clamp, participants will be asked to complete the World Health Organisation—Five Well‐Being Index^25^ (WHO‐5) as a measure of well‐being.

Blood samples will be centrifuged and decanted into microtubes, which will be stored at −80°C. When the study has been completed, serum and plasma samples will be transferred to the Biomarker Core Laboratory at the University of Dundee for processing.

### 4.4 Intervention period

Following visit 3, participants within the CGM alone (control) group will continue with their usual standard of care but with the use of CGM to optimize glycaemic control and aid hypoglycaemia avoidance. Participants within the HIT&CGM group will undergo the HIT programme (Figure 1), three times a week for the 4‐week intervention period plus using the CGM to optimize glycaemic control and avoid hypoglycaemia. In addition, instructions on insulin and carbohydrates adjustment before and after exercise are provided according to recent guidelines.^18^

The aim of the HIT programme is to reach ≥ 90% peak heart rate obtained during the VO_2_ peak assessment. The HIT programme will be carried out on an exercise bike at a local gym convenient for the participant. They will be asked to gently cycle for 5 minutes to warm‐up and then increase the resistance and their revolutions per minute (RPM) for 30 seconds. This is followed by 2‐minute active recovery and repeated 4 times with a 5‐minute cool‐down period at the end (Figure 1).

A member of the research team will attend the first exercise session with each participant to ensure they are familiar with the protocol and able to complete it. Participants in the HIT&CGM group will be given heart rate monitors (*Polar Vantage M and H10; Polar Electro OY*) to use during their exercise sessions to record their heart rate during each session.

During the 4‐week intervention period, there will be a flexible weekly study visit for both study groups. During this visit, CGM data will be downloaded, and advice given to insulin dose adjustment to optimize glycaemic control and aid avoidance of hypoglycaemia. In addition, heart rate data will be downloaded to ensure that all those in the HIT group are reaching their heart rate target (≥90% max) during the HIT sessions.

There will be a 4‐ to 7‐day break at the end of the intervention period prior to the hyperinsulinaemic hypoglycaemic clamp study, visit 8.

Study visit 8 and the hyperinsulinaemic hypoglycaemic clamp method will be identical to visit 3 and will be the final study visit. The study will be completed when the final participant completes their final visit (Figure 2).

### 4.5 Study outcomes

#### 4.5.1 Primary

- The difference in the adrenaline response to hypoglycaemia following the HIT or control interventions

#### 4.5.2 Secondary

- Changes in awareness of hypoglycaemia and symptom scores—Edinburgh Hypoglycaemia Symptom (EHS) score.
- Changes in cognitive function—Digit Symbol Substitution Test (DSST), 4‐Choice Reaction Time (4‐CRT).
- Changes in well‐being—standardized validated questionnaire (The World Health Organisation—Five Well‐Being Index (WHO‐5)).
- Changes in other counterregulatory hormones—glucagon, noradrenaline (NA), lactate and cortisol.

### 4.6 Sample size and power calculation

This is a pilot study and therefore cannot be powered by any previous studies as to the best of our knowledge there has not been a study of its type before.

We will aim to recruit up to 16 participants to each study group with a total of up to 32 participants. This will allow for any dropout during the study.

### 4.7 Recruitment

Participants will be identified using the Scottish Diabetes Research Network (SDRN) Research Register and opportunistically at diabetes outpatient clinics within NHS Tayside.

### 4.8 Randomization

The randomization involved will relate to which intervention group participants are allocated to (HIT&CGM or CGM alone (control)). Randomization will be carried out by an independent individual, at the University of Dundee. Randomization will be done in a GCP‐compliant manner using http://www.randomization.com/.

Randomization will be carried out using a validated Web‐based block randomization generator to ensure that an equal number of participants are assigned to each intervention at baseline. Once the randomization has taken place, the allocation list will be concealed from the CI and CRF until the recruit has been consented. The CRF will be informed of the intervention allocation and will inform the participant once they have consented to take part in the study.

## 5 DATA COLLECTION, MANAGEMENT AND ANALYSIS

### 5.1 Data collection

The data will be collected by the CRF or delegated member of the research team on a paper case report form with subsequent transcription to an electronic case report form. Electronic storage will be in an encrypted form on a password‐protected device. The medical notes will act as source data for medical history, any data relating to general medical history will be filed in the notes.

### 5.2 Statistical methods

For the primary and secondary end points, a mixed model will be used adjusting for baseline as a covariate. *P* < .05 will be considered statistically significant. Statistical analyses will be conducted using IBM SPSS Statistics 22 software.

### 5.3 Data monitoring

A data monitoring committee is not considered necessary as this is a relatively small trial. An experienced Chief Investigator will supervise the CRF. All local protocols will be followed with regard to data monitoring.

## 6 ETHICS AND DISSEMINATION

Ethics approval was obtained by the South East of Scotland Research Ethics Service (18/SS/0160).

The report from the clinical trial will be used for publication and presentation at scientific meetings. The trial investigators will publish the results in writing or via oral presentation.

The trial has been registered with an International Standard Randomised Controlled Trials Number (ISRCTN15373978). It was registered before the enrolment of patients.

Should any protocol modifications arise, these will be decided by the CI and CRF. The protocol will then be reviewed by the trial sponsor, who will decide whether further notification to the relevant authorities is required.

### 6.1 Discussion

Approximately 20%‐30% of all people with type 1 diabetes have impaired awareness of hypoglycaemia,^2^ a condition that negatively impacts on their quality of life and that is associated with significant morbidity. The mechanisms behind the development of impaired awareness of hypoglycaemia are not yet fully understood. There are many features of this phenomenon that are however in keeping with a habituated response, as described by Thompson and Spencer,^9^ and recently reviewed by our group.^4^ Moreover, a cardinal feature of a habituated response is that it can be at least temporarily restored following the introduction of a novel, usually strong, stimulus: dishabituation.^9^ Using a single episode of HIT as a dishabituating stimulus to test this concept, we were recently able to demonstrate improvements in CRR to hypoglycaemia in both rodents^11^ and humans with long‐standing type 1 diabetes.^12^ However, a single episode of HIT is unlikely to have a sustained effect and to fully restore hypoglycaemia awareness in individuals with type 1 diabetes who have been exposed to multiple episodes of hypoglycaemia over many years. In HIT4HYPOS, we will examine in a small, experimental study whether a 4‐week intervention with HIT (12 episodes in total) in addition to standard care for people experiencing recurrent hypoglycaemia will have sustained benefits on symptom awareness and counterregulatory hormone responses to hypoglycaemia. If successful, this will confirm that impaired awareness of hypoglycaemia develops through habituation to recurrent hypoglycaemia and that dishabituation offers a novel, therapeutically attractive approach to improving and potentially treating hypoglycaemia awareness in type 1 diabetes. If this study proves to be successful, we may also need to consider other non–exercise‐related interventions (eg cold exposure) that may prove equally beneficial and applicable to those who have difficulty with exercise.

### 6.2 Limitations

As this is a pilot study, power calculations have not been carried out. A clear limitation is that this is a small single‐centre study. The study is looking at people with type 1 diabetes within the working population, there are eight study visits, two of which are hyperinsulinaemic hypoglycaemic clamp studies (5 hours) as such a dropout rate has been considered, recruiting up to 32 participants to allow for dropouts. We are carrying out a parallel‐group study as it is not known how long any lasting effect from the intervention may have on individuals' response to hypoglycaemia, we are therefore not be able to carry out a randomized crossover study. Although the hyperinsulinaemic hypoglycaemic clamp is the gold standard technique for assessing counterregulatory responses to hypoglycaemia,^19^ the studies are conducted under strictly controlled conditions in a laboratory, and as such, the experience of hypoglycaemia may not fully reflect its real‐world experience.

In summary, HIT4HYPOS is a single‐centre randomized parallel‐group study investigating whether dishabituation with high‐intensity exercise can be used as a novel treatment to improve hypoglycaemia awareness in people with long‐duration type 1 diabetes. This study will provide useful insights in this field where the underlying cause of this common complication is not yet fully understood and treatment options remain limited.

## CONFLICT OF INTEREST

There are no competing interests.

## AUTHOR CONTRIBUTIONS

CMF contributed to research design and drafted the manuscript. DW and ADM contributed to research design and revision of the manuscript. RJM contributed to research design and drafted the manuscript. All authors approved the final version of the manuscript to be published. RJM is responsible for the integrity of the work as a whole.

## Data Availability

Data will be available on request from the author who is guarantor of this work.
